# Source‐anchored, trace‐anchored, and general match score‐based likelihood ratios for camera device identification

**DOI:** 10.1111/1556-4029.14991

**Published:** 2022-02-06

**Authors:** Stephanie Reinders, Yong Guan, Danica Ommen, Jennifer Newman

**Affiliations:** ^1^ Department of Statistics Iowa State University Ames Iowa USA; ^2^ Center for Statistics and Applications in Forensic Evidence Iowa State University Ames Iowa USA; ^3^ Department of Electrical and Computer Engineering Iowa State University Ames Iowa USA; ^4^ Department of Mathematics Iowa State University Ames Iowa USA

**Keywords:** digital cameras, digital evidence, digital images, forensic camera identification, score‐based likelihood ratios and SLR

## Abstract

Forensic camera device identification addresses the scenario, where an investigator has two pieces of evidence: a digital image from an unknown camera involved in a crime, such as child pornography, and a person of interest’s (POI’s) camera. The investigator wants to determine whether the image was taken by the POI’s camera. Small manufacturing imperfections in the photodiode cause slight variations among pixels in the camera sensor array. These spatial variations, called photo‐response non‐uniformity (PRNU), provide an identifying characteristic, or fingerprint, of the camera. Most work in camera device identification leverages the PRNU of the questioned image and the POI’s camera to make a yes‐or‐no decision. As in other areas of forensics, there is a need to introduce statistical and probabilistic methods that quantify the strength of evidence in favor of the decision. Score‐based likelihood ratios (SLRs) have been proposed in the forensics community to do just that. Several types of SLRs have been studied individually for camera device identification. We introduce a framework for calculating and comparing the performance of three types of SLRs — source‐anchored, trace‐anchored, and general match. We employ PRNU estimates as camera fingerprints and use correlation distance as a similarity score. Three types of SLRs are calculated for 48 camera devices from four image databases: ALASKA; BOSSbase; Dresden; and StegoAppDB. Experiments show that the trace‐anchored SLRs perform the best of these three SLR types on the dataset and the general match SLRs perform the worst.

**Highlights**

- SLRs convey the strength of evidence in favor of a match or non‐match for camera identification.
- The three types of SLRs considered achieve low rates of misleading evidence on the dataset.
- Trace‐anchored SLRs outperform source‐anchored and general match SLRs on the dataset.

## 1 INTRODUCTION

Digital image forensics is a branch of forensic science that analyzes digital photographs and videos. Like many other areas of pattern evidence identified in the 2009 landmark report by the National Research Council [1], multimedia analysis was identified as lacking in probabilistic and statistical foundations. This absence of sound scientific methods provides challenges to meet the Daubert standard established by the court case Daubert v. Merrell Dow Pharmaceuticals [2] and can compromise the probative value of the evidence. Evidentiary strength of forensic findings relies on rigorous and peer‐reviewed research experiments, where the reliability and validity of the analysis has been tested so it will withstand increasing scrutiny in the courts [3]. *Score‐based likelihood ratios* (SLRs) provide one method for quantifying the probative value of a piece of evidence and are an area in which digital pattern evidence is building its repertoire of evidence‐based research findings. In this paper, we develop a framework for calculating three types of SLRs to quantify the weight of evidence for the digital image forensic problem of *camera device identification*, where the goal is to identify a particular camera device (as opposed to camera model) that captured a questioned image.

In pattern evidence analysis, the investigator is often confronted with a *source identification* problem. The investigator has two impressions, one impression *E*_u_ from the crime scene where *E*_u_ is from an *unknown source*. The other impression *E*_k_ is directly acquired from a *specific known source*, related to the person of interest (POI). The investigator asks: how likely is it that both impressions originate from the specific known source? How likely is it that the crime scene impression does not originate from the specific known source? These questions are often expressed as two competing, specific‐source hypotheses [4].

$$\begin{array}{c} \begin{array}{c} H_{p}:\text{the impression}\;E_{u}\;\text{originated from the specific}\text{known source that created}\;E_{k},\\ H_{d}:\text{the impression}\;E_{u}\;\text{originated from}\;a\;\text{different source}\text{than the specific known source.} \end{array} \end{array}$$

In practice, the prosecution and the defense do not specify these hypotheses, but we will use standard nomenclature and refer to *H*_p_ as the prosecution’s hypothesis and *H*_d_ as the defense’s hypothesis. An investigator faced with a camera device identification problem might have a digital image *I*_u_ that contains child pornography and a camera fingerprint *F*_k_ estimated from images of innocuous content from a POI’s camera. (We use a capital letter *I* to denote images and a capital letter *F* to denote camera fingerprints. The subscripts denote the camera that created the image or fingerprint, the letter u stands for unknown camera and the letter k stands for the POI’s camera, which is also called the specific known device.) The prosecution’s hypothesis is that the child pornography image originated from the POI’s camera that also created the camera fingerprint, while the defense’s hypothesis is that the child pornography image did not originate from the POI’s camera.

Most camera device identification methods rely on a camera sensor property called photo‐response non‐uniformity (PRNU) [5]. The measured response of a camera sensor array to incoming photons slightly varies from pixel to pixel due to the manufacturing process and imperfections in the photodiode. In principle, the spatial placement of these variations from the mean response of the array of pixels provides an identifying characteristic, or fingerprint, of the camera. We will use the terms PRNU and camera fingerprint interchangeably.

**FIGURE 1:**
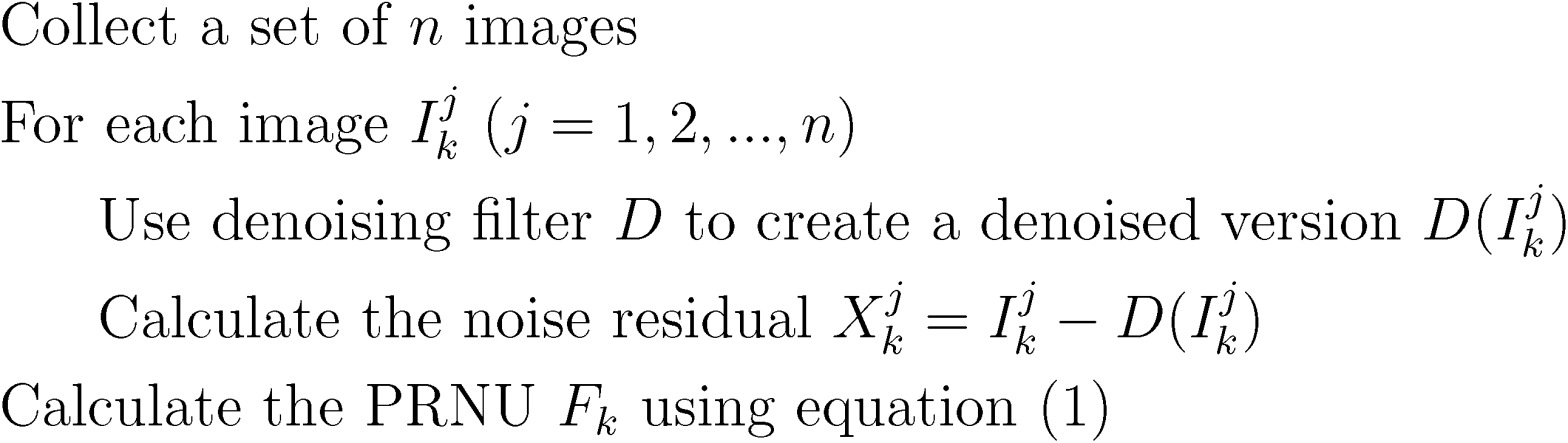
Summary of the PRNU estimation algorithm presented in [6, 7]

In Figure 1, we summarize the algorithm presented in [6, 7] to estimate the camera fingerprint *F*_k_ of device *C*_k_, which is given by equation

(1) $$F_{k}=\frac{\sum_{j=1}^{n}X_{k}^{j}I_{k}^{j}}{\sum_{j=1}^{n}{\left(I_{k}^{j}\right)}^{2}},$$

where $I_{k}^{j}$ (*j* = 1, 2, …, *n*) are images from *C*_k_ and $X_{k}^{j}$ (*j* = 1, 2, …, *n*) are noise residuals of the images calculated by subtracting a denoised version of the image from the image itself. The image $I_{k}^{J}$, the noise residual $X_{k}^{j}$, and the fingerprint *F*_k_ in Equation (1) can all be represented as matrices of pixel values and multiplication is performed element‐wise. Note that we use slightly different notation than that in [6, 7]: we use a subscript to denote the camera device to which an image belongs.

An investigator can estimate the true PRNU of a camera using the above algorithm and a set of images from the POI’s camera. If the investigator has one single image, then the PRNU estimate is simply the noise residual of the single image. An assumption made of the extracted PRNU is that it is unique for a camera sensor. A recent paper [8] presents results that show that recent advances in the image processing pipeline such as customized HDR algorithms can cause small similarities in the camera fingerprints from different cameras of the same model. They show that these small similarities can slightly increase the rate of false positives between different cameras of the same model. We invite readers to pursue open questions that arise from this recent publication.

After estimating the camera fingerprint *F*_k_ from device *C*_k_ a questioned image *I*_u_ from unknown device *C*_u_ is compared with *F*_k_ using a (dis)similarity score Δ. The value of interest is

(2) $$\delta =\Delta \left(X_{u},I_{u}F_{k}\right),$$

where multiplication between *I*_u_ and *F*_k_ is performed element‐wise (See Ref. [6] for an explanation of why the noise residual is compared to the product of the image and the camera fingerprint). Early camera device identification works used the sample correlation as the similarity score [5, 6, 9, 10]. Later the peak‐to‐correlation energy (PCE) replaced sample correlation because the PCE is robust against a periodic signal called *linear pattern* that created problems for statistical models that used sample correlation [11]. Most previous work in camera device identification focuses on developing what we term a *universal detector* [7, 9, 11, 12, 13, 14, 15] where the authors aim to create a single system that works for any questioned image and camera device. Over the years, universal detectors that use the PRNU have been tested against cropping and scaling [13], gamma correction and denoising [6], compression [14], lens distortion [16], and contrast enhancement, histogram equalization, and white balance [15]. Most previous work determines if a questioned image *I*_u_ came from a specific known camera *C*_k_ by comparing the value of interest *δ* = Δ(*X*_u_, *I*_u_ *F*_k_) from Equation (2) to an ad‐hoc decision threshold *t*, where *t* is typically chosen based on a constructed set of similarity scores between an image and a camera fingerprint known to be from the same camera (matching) and an image and a camera fingerprint known to be from different cameras (non‐matching). In the universal detector approach, the researchers' goal is to create a single detector and decision threshold *t* that can be applied to any image and any camera device. Of particular interest to us, the universal detector approach addresses the common‐source hypotheses, which are less pertinent to the decision‐makers in criminal justice trials than the specific‐source hypotheses (see Ref. [4] for more information on the common/specific‐source problems). Furthermore, the universal detector methods aim to *classify* pairs of images as either matching or non‐matching, using pattern recognition and classification methods, such as linear discriminant analysis, to define the decision threshold. This results in a binary decision that gives no information about the strength of the evidence in favor of that decision. These methods differ from the likelihood ratio‐style approaches, which aimed to *quantify* the probability of observing the evidence under two competing hypotheses.

Likelihood ratios (LRs) are used in single source DNA analysis [17] and glass fragment analysis [18, 19]. An LR for pattern evidence is defined as the ratio of the likelihoods of observing both impressions under hypotheses *H*_p_ and *H*_d_. More specifically, for an impression *E*_u_ from a crime scene and an impression *E*_k_ from a source related to the POI, the LR would be written

(3) $$\mathrm{LR}=\frac{P\left(E_{u},E_{k}|H_{p}\right)}{P\left(E_{u},E_{k}|H_{d}\right)},$$

where *P*(·) is a joint probability density (or mass) function (PDF) [20]. The PDF in the numerator of the LR describes the likelihood of observing both impressions *E*_u_ and *E*_k_ if they originated from the specific known source and the PDF in the denominator would be the likelihood of observing both impressions *E*_u_ and *E*_k_ if they were a “random match” and *E*_u_ came from a different source than the specific known source. To formulate an LR as in Equation (3), the investigator needs an applicable set of measurements, often called features, where the variability between the features of two impressions from the same source can be distinguished, with high accuracy, from the variability between the features of two impressions from different sources. The representation of many pattern evidence data is often high‐dimensional and complex, making such sets of features extremely challenging to identify. Alternate methods for assessing the weight of the evidence are being explored in various forensic fields, including machine learning with paired feature differences [21] and SLRs [22].

To our knowledge, full‐fledged likelihood ratios have not yet been implemented in camera device identification, but Qiao et al. [23, 24] employed a related method, a likelihood ratio test (LRT), for camera device identification to determine which of two camera devices *C*_1_ or *C*_2_ captured a questioned image *I*_u_ from an unknown source. They formulate the problem as a classification problem and consider the following two hypotheses:

$$\begin{array}{c} H_{1}:I_{u}\;\text{originated from device}\;C_{1}\\ H_{2}:I_{u}\;\text{originated from device}\;C_{2}. \end{array}$$

Note that we use our own notation here to be consistent with the rest of this paper. The authors also address the question of which device from a set of *n* devices *C*_1_, *C*_2_, …, *C*_*n*_ took questioned image *I*_u_ by performing an LRT for each possible pair of devices and declaring the device identified by the most LRTs to be the device that captured the *I*_u_ [23]. If a case arises in practice where a questioned image is known to have been taken by one of two devices, the likelihood ratio that Qiao et al. treat as the test statistic in their likelihood ratio test could potentially quantify the strength of evidence. Because their intent was to solve a multi‐classification problem by determining which of *n* classes (cameras) the question image *I*_u_ came from, this framework does not readily provide a means of quantifying the strength of the evidence when the pool of potential sources of *I*_u_ is larger than two devices. LRs still remain elusive for camera device identification.

SLRs have appeared in a variety of forensic fields when LRs are unavailable: glass fragments and shoe impressions [22]; handwriting [25]; MDMA tablets [26]; fingerprints [27]; speaker recognition [28]; and facial recognition [29]. SLRs use a similarity score Δ to measure the similarity (or dissimilarity) of two pieces of evidence *E*_u_ and *E*_k_ and then calculate the likelihood of obtaining the score Δ(*E*_u_, *E*_k_) under hypotheses *H*_p_ and *H*_d_. An SLR is then the ratio of these two likelihoods. A reference set of *matching* scores between two pieces of evidence known to come from the same source are used to estimate the PDF in the SLR numerator. The PDF in the denominator is estimated from a reference set of *non‐matching* scores between two pieces of evidence known to come from different sources. We consider three methods presented by Hepler et al. [25] – trace‐anchored, source‐anchored, and general match – for constructing non‐matching scores to estimate the SLR denominator for handwriting evidence. Consider the scenario where investigators have a questioned handwritten document *E*_u_ and a handwriting sample *E*_k_ from a POI. They specify an alternative population of possible writers. Researchers and practitioners are still in disagreement about how alternative populations should be constructed [30, 31]. The trace‐anchored method considers similarity scores between the questioned document *E*_u_ and handwriting samples from writers in the alternative population. Hepler et al. [25] define the trace‐anchored SLR as

(4) $${\mathrm{SLR}}_{\text{trace}}=\frac{P\left(\Delta \left(E_{u},E_{k}\right)|E_{k},H_{p}\right)\;}{P\left(\Delta \left(E_{u},E_{k}\right)|E_{u},H_{d}\right)\;}.$$

Neumann and Ausdemore [32] present an alternative definition of a trace‐anchored SLR:

$${\mathrm{SLR}}_{\text{trace}}=\frac{P\left(\Delta \left(E_{u},E_{k}\right)|E_{u},H_{p}\right)\;}{P\left(\Delta \left(E_{u},E_{k}\right)|E_{u},H_{d}\right)\;}.$$

In this case, both the numerator and denominator condition on the evidence from the unknown source, unlike Equation (4) that anchors on the evidence from the known source in the numerator and the evidence from the unknown source in the denominator. Neumann and Ausdemore state an SLR that anchors on the evidence from the unknown source in the numerator is not useful in practice because the investigator would need other objects from the same unknown source as *E*_u_ to estimate the numerator. Additionally, Neumann and Ausdemore point out that because the trace‐anchored SLR defined by Hepler et al. anchors on two different objects in the numerator and denominator it is highly unlikely that it will converge to the desired Bayes factor. However, this is only a drawback from a Bayesian perspective and the validity of this method for other statistical frameworks has yet to be explored.

The source‐anchored method considers similarity scores between the POI’s handwriting sample *E*_k_ and handwriting samples from writers in the alternative population. The source‐anchored SLR is defined

(5) $${\mathrm{SLR}}_{\text{source}}=\frac{P\left(\Delta \left(E_{u},E_{k}\right)|E_{k},H_{p}\right)\;}{P\left(\Delta \left(E_{u},E_{k}\right)|E_{k},H_{d}\right)\;}.$$

Neumann and Ausdemore [32] criticize this approach for being incoherent from a Bayesian perspective, but Garton [33] takes a different viewpoint.

The general match method considers similarity scores between handwriting samples from pairs of different writers randomly selected from the alternative population. The general match SLR is defined

(6) $${\mathrm{SLR}}_{\text{general}}=\frac{P\left(\Delta \left(E_{u},E_{k}\right)|E_{k},H_{p}\right)\;}{P\left(\Delta \left(E_{u},E_{k}\right)|H_{d}\right)\;}.$$

Like the trace‐anchored SLR, the general match SLR also does not anchor on the same object in the numerator and denominator. Again, this is problematic from the Bayesian perspective, but further research is needed to ascertain whether this is an issue from the perspective of other statistical frameworks. Hepler et al. [25] demonstrate that these three SLR types can lead to different conclusions on the same evidence. Because of this, the method investigators use to build their reference set of non‐matching scores is extremely important.

In many situations, SLRs from Equations (4), (5), (6) are easier to apply than LRs from Equation (3) because similarity scores reduce the dimensionality of the problem. Instead of needing to fit probability distributions in high dimensions, the investigator only needs to fit distributions to lower dimensional similarity scores. One drawback of SLRs is that unlike LRs, they do not account for rarity. For example, if a witness told the police the color, make, and model of the car that fled the crime scene and a person of interest owns a car of the same color, make, and model, an LR would take into account the rarity of that color, make, and model in the general population of cars (e.g., a red Ferrari Portofino is rarer than a black Toyota Corolla). In contrast, an SLR does not consider the rarity of the car involved (e.g., two red Ferrari Portofino cars are just as similar in color, make and model as two black Toyota Corolla cars). Another drawback of SLRs is that when “pairwise comparison” methods are used, dependency is introduced into the resulting score data. Meaning that any two similarity scores that were created using the same object as one item in the pair will be dependent (e.g., Δ(*I*_1_, *I*_2_) and Δ(*I*_1_, *I*_3_) are dependent because they both involve item *I*_1_). Unfortunately, no one knows how to fix this problem yet. Because LRs are as of yet unavailable for camera device identification when more than two devices are considered, despite the limitations, SLRs are the only available alternative.

SLRs have been applied to the digital image forensic problem of *camera source identification*, where the source being identified is a particular camera device [34, 35]. Nordgaard and Höglund [34] introduce the framework for calculating source‐anchored SLRs, one of the available types of SLRs. They perform simulation studies in the case where a questioned image came from one of two cameras, and they discuss how their method could be applied to a larger set of cameras. van Houten et al. [35] addressed the scenario where an investigator knows the make and model of the camera that took a questioned image and wishes to determine which device out of a set of 9 or 10 devices of that make and model captured the image. They present specific‐source hypotheses, but they construct SLRs that address common‐source hypotheses. The difference between the numerators of a common‐source SLR and a specific‐source SLR lies in the construction of the reference sets of matching scores used to estimate the numerators' PDFs [31]. The reference set of matching scores for a common‐source SLR consists of matching scores from devices in the alternative device population in addition to matching scores from the specific known device. The specific‐source SLR uses matching scores only from the specific known device. The denominator of a common‐source SLR is the same as the general match denominator for a specific‐source SLR. van Houten et al. performed experiments on two camera models: the Motorola V360 mobile phone camera (10 cameras) and the Sony DSC‐S500 camera (nine cameras). Reinders [36] and Reinders et al. [37] adapted the method Hepler et al. used to calculate trace‐anchored SLRs for the camera device identification problem. We present an extended investigation of the use of SLRs for camera device identification beyond the current published literature and develop a framework for calculating all three available types of specific‐source SLRs with a larger dataset of 48 camera devices from 26 distinct camera models. We do not compare our method of computing source‐anchored SLRs to those in the literature since other authors tailored their methods to small database sizes, and we designed our methods to work with large databases.

**FIGURE 2:**
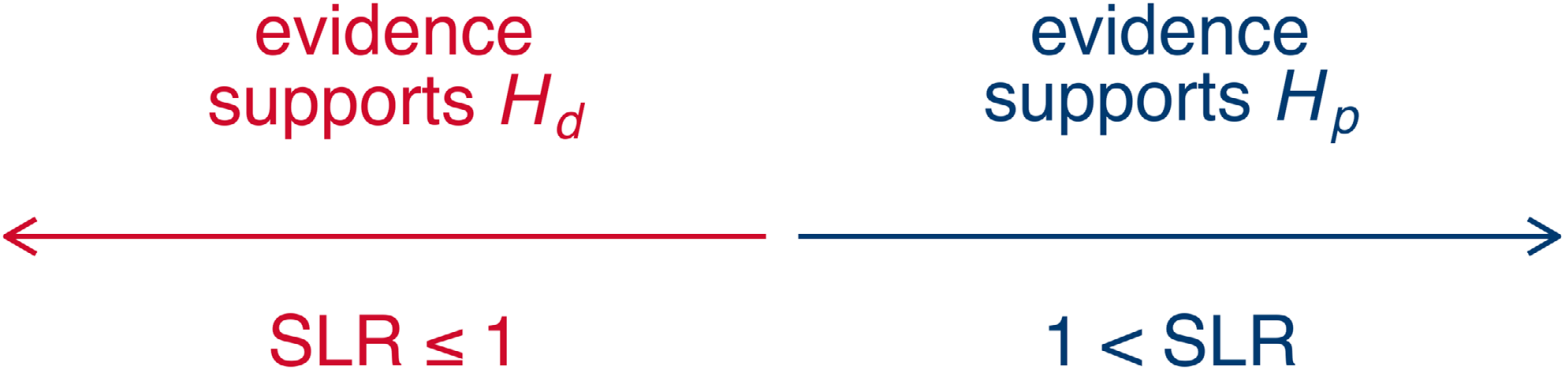
Basic interpretation of SLR values

As mentioned before, SLRs quantify the strength of evidence in favor of one of the hypotheses over the other. More specifically, if an SLR is greater than 1 it shows support for *H*_p_ rather than *H*_d_ and the larger the SLR the stronger the support for *H*_p_. On the other hand, an SLR value less than or equal to 1 supports *H*_d_ rather than *H*_p_ and the smaller the SLR the stronger the support for *H*_d_. Figure 2 illustrates this.

Section 2 describes the proposed framework for calculating the three types of SLRs for camera device identification. Section 3 explains the results obtained from applying the proposed methods to a dataset of 48 camera devices. Section 4 discusses the strengths and limitations of the proposed methods and the implications of the findings for future work.

## 2 METHODS

We present a framework for calculating three types of specific‐source SLRs [25] – trace‐anchored, source‐anchored, and general match – for the camera device identification problem. We demonstrate this framework on an image dataset from 48 camera devices representing 26 distinct camera models. Building upon previous work, our framework offers a more comprehensive application of SLRs for camera device identification.

We start by describing the scenario we consider. Then we formulate two competing hypotheses *H*_p_ and *H*_d_ for this scenario. We specify the alternative camera devices and image data that we use in our analyses. We estimate camera fingerprints from the POI’s camera and each of the alternative devices. Then we calculate a similarity score *δ* between the questioned image and the camera fingerprints from the POI’s camera. To estimate the probabilities of observing the score *δ* under each hypothesis, we build reference sets of matching and non‐matching scores using image data from the alternative camera devices. Finally, we construct three types of SLRs as the ratio between probability of observing *δ* under *H*_p_ (same source) and the probability of observing *δ* under *H*_d_ (different source).

We consider the scenario where an investigator has two pieces of evidence: a digital image from an unknown camera device that was involved in a crime; and a camera fingerprint from a POI’s camera device. Our method requires that the investigator has access to one or more images that are known to have originated from the POI’s camera that can be used to estimate a camera fingerprint. Instead of using the generic labels *E*_u_ and *E*_k_ for evidence as in the previous section, here we denote a questioned image of unknown source as *I*_u_ to make clear that the evidence is an image. We use *F*_k_ to denote a camera fingerprint from the specific known source, the POI’s camera, and we use *C*_k_ to refer to the POI’s camera. We construct the following two competing hypotheses:

$$\begin{array}{c} \begin{array}{c} H_{p}:\;\text{questioned image}\;I_{u}\;\text{and camera fingerprint}\;\;F_{k}\;\text{both originated from camera device}\;C_{k}\;\\ H_{d}:\;\text{camera fingerprint}\;F_{k}\;\text{originated from camera device}\;\;C_{k},\mathrm{but}\;\text{questioned image}\;I_{u}\;\mathrm{did}\;\text{not}. \end{array} \end{array}$$

The goal then is to construct score‐base likelihood ratios to evaluate the strength of the evidence regarding these specific‐source hypotheses.

Our experiments use image data from four digital image databases: Alaska version 1 (R. Cogranne, Q. Giboulot, P. Bas, personal communication, December 1, 2019), BOSSbase [38], Dresden (Dressden Image Database, T. Gloe, R. Bohme, personal communication, December 1, 2019), which is described in [39], and StegoAppDB [40, 41]. Because this work is the first implementation of our proposed framework, we chose to restrict our experiments to RAW, auto‐exposure images that we converted to TIFF format using Adobe Photoshop’s Image Processor without LZW compression or resizing. We use photos taken in landscape orientation only and ignore devices with fewer than 100 such images. Restricting to auto‐exposure eliminates the effect of manual camera exposure, which may affect the PRNU accuracy. Using the RAW image data and converting to TIFF eliminates the effect of compression quality from JPEG images that can also affect accuracy. Landscape photos avoid calculation of rotation of the device to perform the “best” fit, another computational issue we put aside. Finally, we use 512 × 512 sub‐images cropped from the center of each photo, rather than the entire photo itself. This avoids the computationally expensive alignment process to compare images of different sizes, as would be necessary in real‐world case scenarios. These choices limit the effect of other complicating factors that potentially affect accuracy, so that analysis of SLR scores can avoid complicating factors. Future work should investigate these and other factors for their impact to accuracies.

A total of 48 devices from all four databases had at least 100 RAW, auto‐exposure, landscape‐oriented images, so these are the devices that we use in our experiments. Of those 48 devices, 23 are digital still cameras, 24 are mobile phones, and one is a tablet. The 48 devices represent 26 distinct camera models and 16 of the models have at least two devices. Ideally, we would have a much larger set of devices, but this is the largest set of images that we could find where the ground truth of the camera device has been authenticated. We randomly select 100 images from each of the 48 devices and pre‐process the images by converting the RAW images to TIFF in Photoshop using the Image Processor with no LZW compression or resizing. Then we center‐crop the images to 512 × 512 and save them as PNG in MATLAB. (Images can be cropped in Python using the Python Imaging Library or similar libraries.) We split the sample of 100 images from each of the 48 devices into a training set of 80 images and a set of 20 testing images, which serve as our questioned images. We have 20 × 48 = 960 questioned images in total.

Camera fingerprints are estimated from each of the 48 camera devices. (MATLAB and Python implementations of fingerprint estimation are available at [42]. We used the MATLAB code.) Each device *C*_*i*_ has *n* = 80 training images $I_{i}^{1},I_{i}^{2},\ldots ,I_{i}^{n}$. (The subscript denotes the camera, and the images are numbered in the superscript.) A denoising filter *D* is used to extract a noise residual from each image: $X_{i}^{j}=I_{i}^{j}-D\left(I_{i}^{j}\right)$ for *j* = 1, 2, …, *n*. The *n* = 80 training images are divided into 8 folds of 10 images each. Camera fingerprint $F_{i}^{1}$ calculated with Equation (1) and all training images except those in fold 1, camera fingerprint $F_{i}^{2}$ is estimated from all training images except those in fold 2, and so on. This results in 8 camera fingerprints from device *C*_*i*_.

We need a way to measure the similarity (or dissimilarity) between the questioned image *I*_u_ and the camera fingerprint *F*_k_ from the POI’s device. In other words, we need to choose which similarity score to use in Equation (2). We used peak‐to‐correlation energy (PCE) in our initial experiments, but we found that the large variance (on the order of 10^6^) of the observed PCE scores produced many unstable SLR values where the numerator of the SLR is tiny and the denominator is zero. We found that for our dataset the sample correlation has much smaller variance (on the order of 10^−3^) and thus produced more stable results. We chose to use the correlation distance, which is defined as one minus the sample correlation. For $X,Y\in {\mathbb{R}}^{n}$ the correlation distance is

$$1-\frac{{\left(X-\bar{X}\right)}^{\prime }\left(Y-\bar{Y}\right)}{\sqrt{{\left(X-\bar{X}\right)}^{\prime }\left(X-\bar{X}\right)}\sqrt{{\left(Y-\bar{Y}\right)}^{\prime }\left(Y-\bar{Y}\right)}},$$

where $\bar{X}=\frac{1}{n}\sum_{i=1}^{n}\;X_{i}$ and $\bar{Y}=\frac{1}{n}\sum_{i=1}^{n}Y_{i}$ . The correlation distance between two images *X* and *Y* with dimensions *m* × *n* can be calculated by first converting the images to vectors of length *mn*. The noise residual *X*_u_ = *I*_u_ − *D*(*I*_u_) is obtained by subtracting a denoised version of the image, created with denoising filter *D*, from the image itself. Then the investigator calculates the correlation distance between the questioned image *I*_u_ and the camera fingerprint *F*_k_ from the POI’s camera using Equation (2) where multiplication between *I*_u_ and *F*_k_ is performed element‐wise and *X*_u_ and *I*_u_ *F*_k_ are first converted to vectors.

A trace‐anchored, source‐anchored, and general match SLR is calculated for each questioned image *I*_u_ and each of the 48 devices set as the specific known device *C*_k_ in turn. For a given questioned image *I*_u_ and a given specific known device *C*_k_ the correlation distance is calculated between a noise residual *X*_u_ of *I*_u_ and the product (element‐wise) $I_{u}F_{k}^{j}$ (for *j* = 1, …, 8) and the results are averaged:

(7) $$\delta =\frac{1}{8}\sum_{j=1}^{8}\Delta \left(X_{u},I_{u}F_{k}^{j}\right).$$

By taking the average score over the eight fingerprints, we are adapting the subsampling algorithm used by Hepler et al. [25] for estimating the numerator distribution for the device identification problem by creating “pseudo camera fingerprints.”

We build reference sets of known matching scores and three sets of non‐matching scores – trace‐anchored, source‐anchored, and general match – to estimate the probability of obtaining the score *δ* under each hypothesis. The reference set of matching scores is used to estimate the probability of obtaining the score *δ* if *I*_u_ and *F*_k_ originated from the same camera. A matching score is calculated as the correlation distance between the *j*‐th fingerprint $F_{k}^{j}$ and the noise residual $X_{k}^{\ell ,j}$ of image $I_{k}^{\ell ,j},$ which is the ℓ‐th image in fold *j*.

$$\text{Matching scores}:\;\Delta \left(X_{k}^{\ell ,j},I_{k}^{\ell ,j}F_{k}^{j}\right)\;\text{for}\;\ell =1,\ldots ,10\;\text{and}\;j=1,\ldots ,8.$$

**FIGURE 3:**
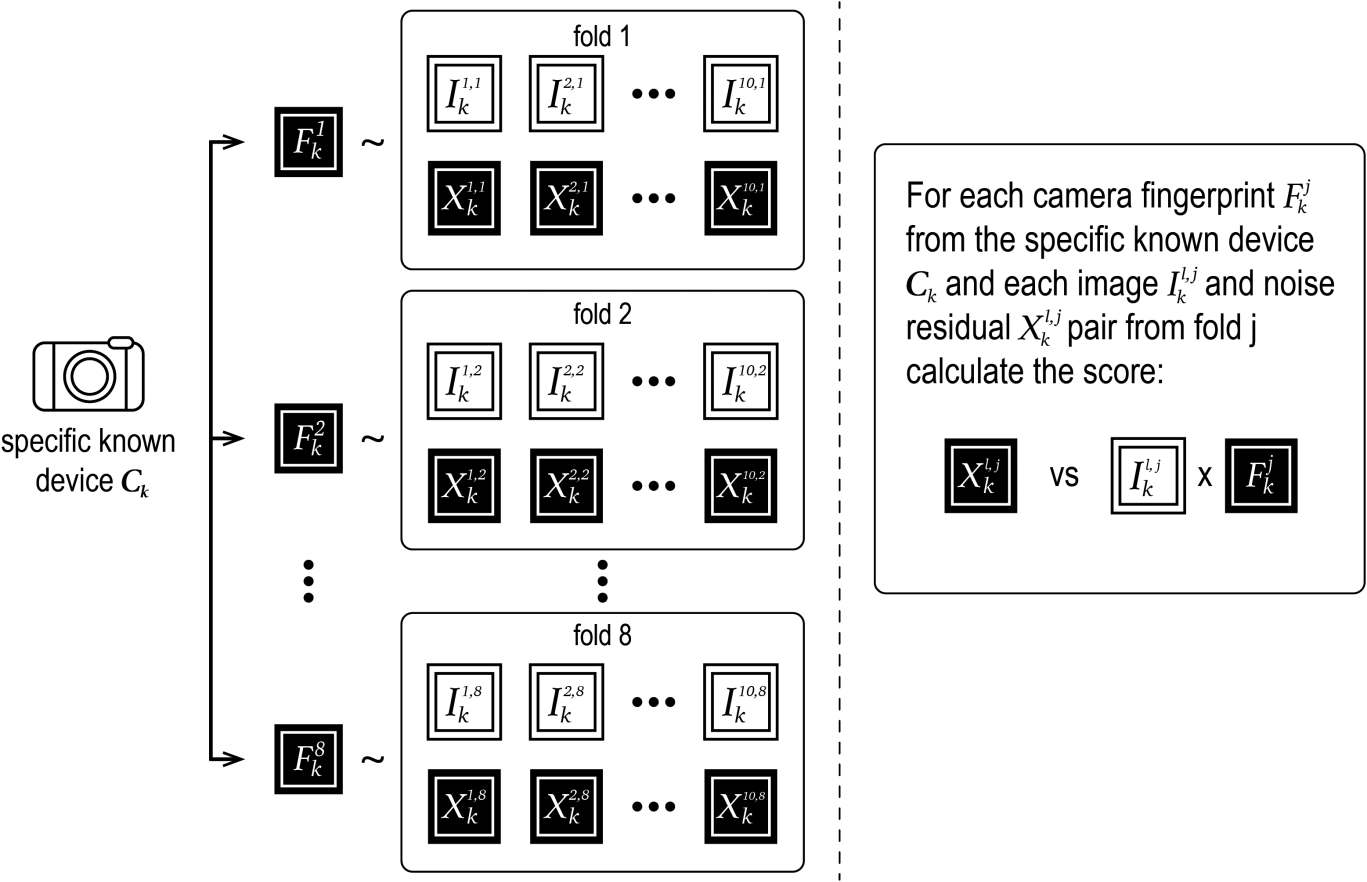
Calculating matching scores for specific known device *C*_k_. Each camera fingerprint $F_{k}^{j}$ was estimated from all training images except those in fold *j*. because the images in fold *j* were not used to estimate fingerprint $F_{k}^{j}$, matching scores are calculated between fingerprint $F_{k}^{j}$ and the images and corresponding noise residuals in fold *j*

Figure 3 illustrates the calculation of matching scores. In total, we calculate 8 × 10 = 80 matching scores for device *C*_k_. Note that scores calculated using the same fingerprint or the same noise residual and image are dependent, so we do not have 80 independent scores. Also, note that matching scores do not use the question image *I*_u_.

Non‐matching scores require a set of alternative camera devices. In our experiments, we set aside specific known device *C*_k_ and treat the other 47 devices from our dataset as the set of alternative camera devices. To calculate a trace‐anchored non‐matching score, we fix the questioned image *I*_u_ and calculate the correlation distance between its noise residual *X*_u_ and a camera fingerprint *F*_a_ from an alternative device *C*_a_.

$$\text{Trace}‐\text{anchored non}‐\text{matching score}:\;\Delta \left(X_{u},I_{u}F_{a}\right).$$

**FIGURE 4:**
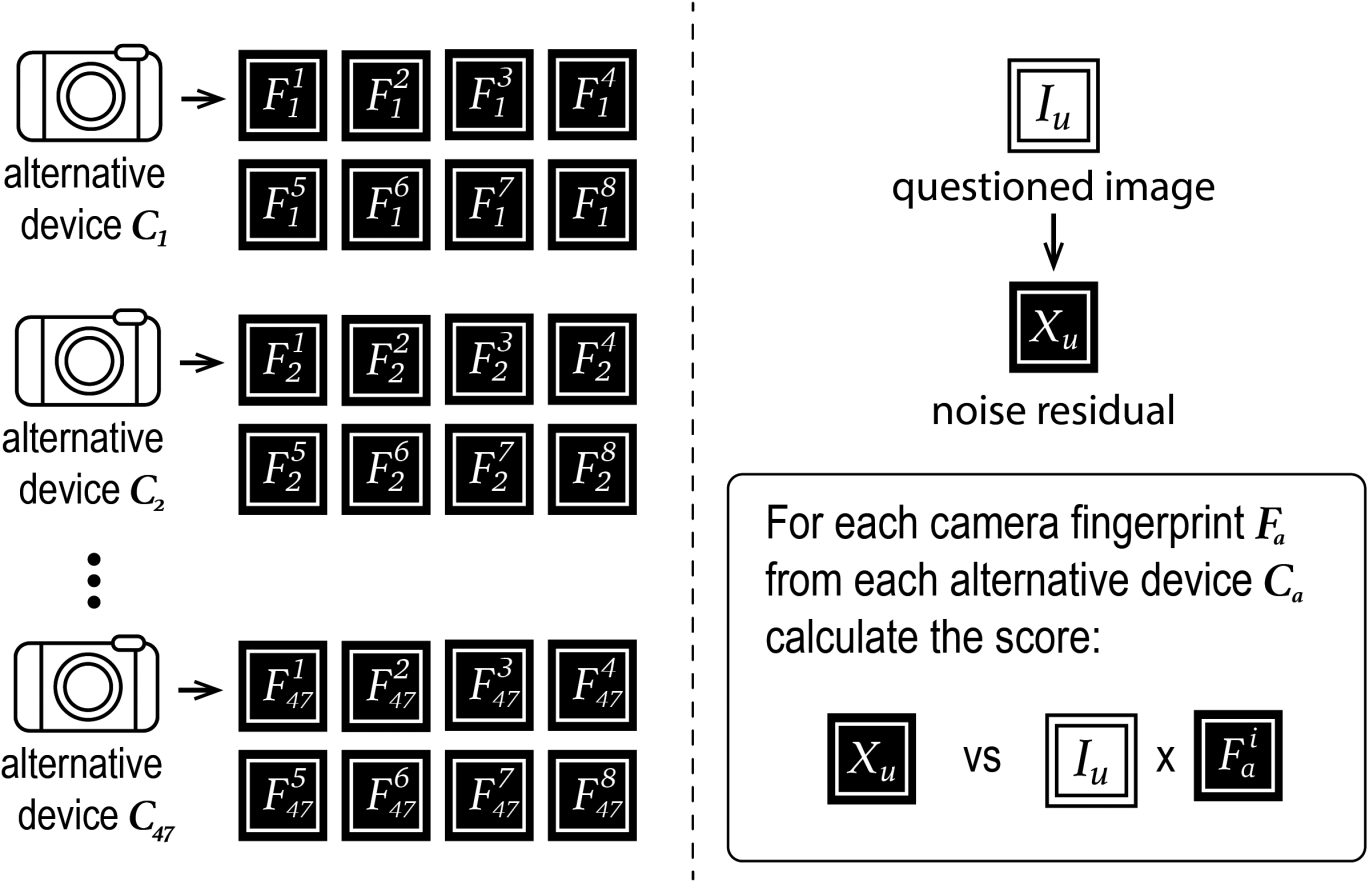
Calculating trace‐anchored non‐matching scores for questioned image *I*_u_ and specific known device *C*_k_

As illustrated in Figure 4, we calculate trace‐anchored non‐matching scores between questioned image *I*_u_ and each of the eight fingerprints from each of the 47 alternative devices. This results in 8 × 47 = 376 trace‐anchored non‐matching scores for questioned image *I*_u_. Because some of these scores were calculated with either the same fingerprint or the same noise residual and image these scores are not independent. Notice that trace‐anchored scores do not use any information from the POI’s device *C*_k_. Also, while we know that *C*_a_ and the POI’s camera *C*_k_ are not the same device, the questioned image *I*_u_ could have originated from *C*_a_. However, our hypotheses ask whether *I*_u_ originated from a device other than *C*_k_ but do not identify the alternative source of the image. If the investigator wants to evaluate the probability that *I*_u_ originated from device *C*_a_, then new hypotheses should be constructed and a new SLR with *C*_a_ in place of *C*_k_ as the specific known device will be calculated.

A source‐anchored non‐matching score is calculated between a fingerprint *F*_k_ of specific known device *C*_k_ and the noise residual *X*_a_ of a training image *I*_a_ from alternative device *C*_a_.

$$\text{Source}‐\text{anchored non}‐\text{matching score}:\;\Delta \left(X_{a},I_{a}F_{k}\right).$$

**FIGURE 5:**
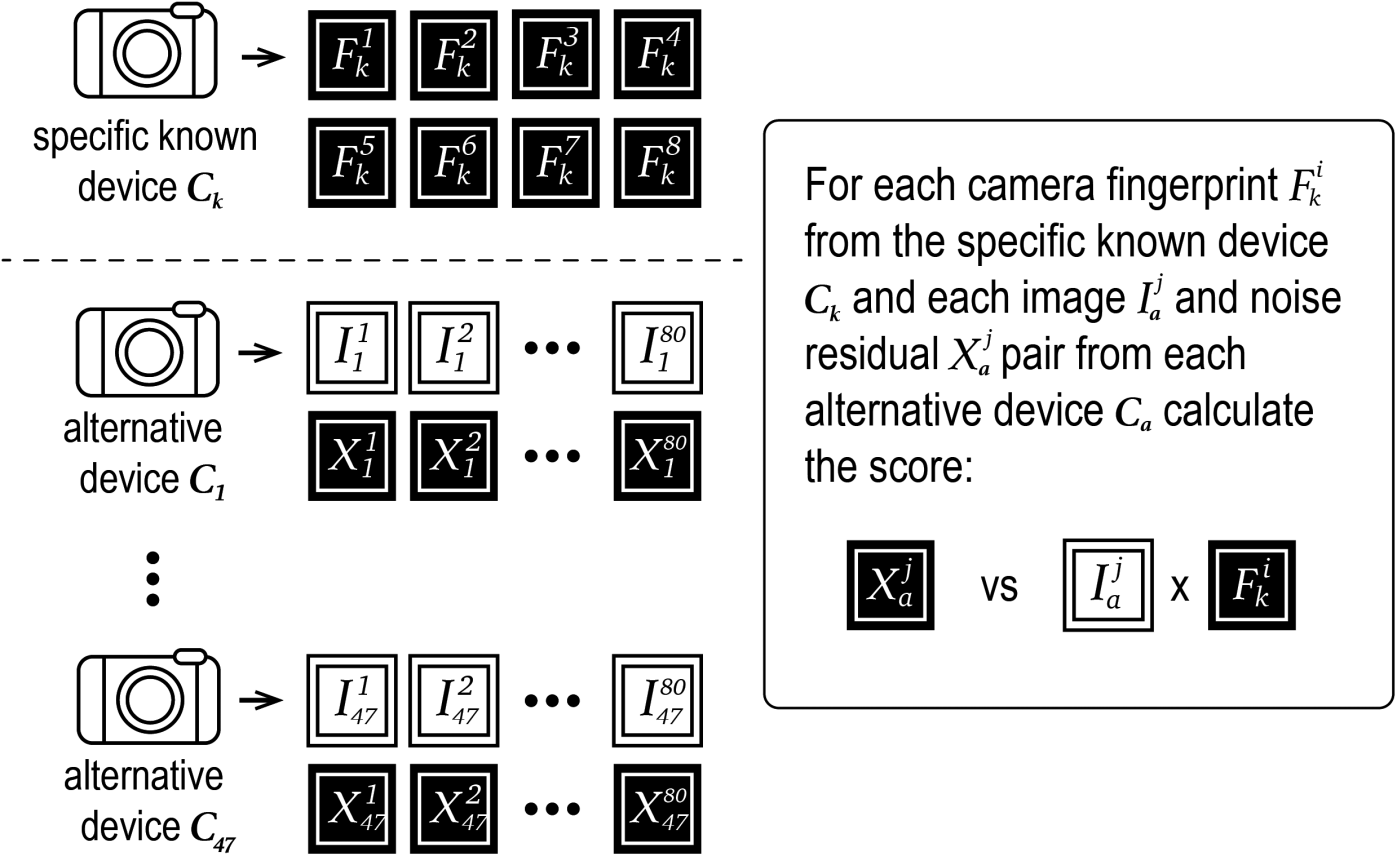
Calculating source‐anchored non‐matching scores for specific known device *C*_k_

We calculate source‐anchored non‐matching scores between each of the 8 camera fingerprints from *C*_k_ and each of the 80 training images from each of the 47 alternative devices for a total of 8 × 80 × 47 = 30,080 scores. Many of these scores are dependent because they are calculated from either the same fingerprint or the same noise residual and image. Figure 5 illustrates the calculation of these scores. Note that the questioned image *I*_u_ is not considered in the source‐anchored scores.

The last type of non‐matching scores are general match non‐matching scores. To calculate one of these scores we randomly select two different devices *C*_1_ and *C*_2_ from the set of 47 alternative devices. Then we calculate the correlation distance between the noise residual *X*_1_ of a training image *I*_1_ from one of the devices with a camera fingerprint *F*_2_ from the other device.

$$\text{General match non}‐\text{matching scores}:\;\Delta \left(X_{1},I_{1}F_{2}\right)\;\text{and}\;\Delta \left(X_{2},I_{2}F_{1}\right).$$

**FIGURE 6:**
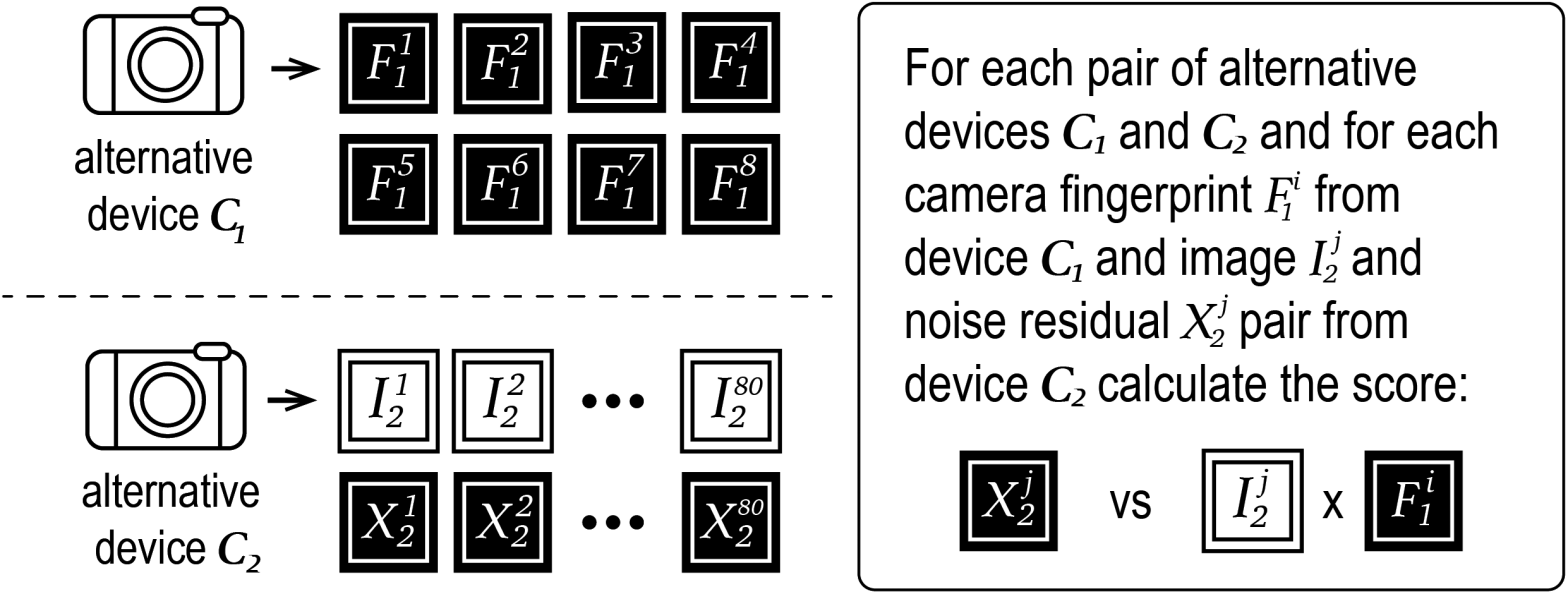
Calculating general match non‐matching for specific known device *C*_k_

We also calculate Δ(*X*_2_, *I*_2_ *F*_1_) where *X*_2_ is the noise residual of a training image *I*_2_ from device *C*_2_ and *F*_1_ is a camera fingerprint from device *C*_1_ because Δ(*X*_1_, *I*_1_ *F*_2_) is not generally equal to Δ(*X*_2_, *I*_2_ *F*_1_). Figure 6 shows that we calculate a general match non‐matching score between the 80 training images of one device and the 8 camera fingerprints of the other device for each pair of alternative devices. This results in 47 × 46 × 80 × 8 = 1,383,680 general match non‐matching scores. Again, many of these scores are dependent because they were calculated from either the same fingerprint or the same noise residual. Notice that the general match non‐matching scores do not use the questioned image or the specific known device.

We do not know the true PDFs of matching and non‐matching scores, so we fit PDF estimates to each set of scores and use these estimates to construct the SLR. We acknowledge that there are several possible methods for estimating these PDFs, including both parametric and non‐parametric options. Nordgaard and Höglund [34] use a parametric method due to the relatively low amount of background information (they only have two camera devices to use for comparison). In contrast, we have much more background information (hundreds of images from 48 camera devices), so we chose to explore a non‐parametric method. We use kernel density estimation to fit PDFs *f*_m_, *f*_trace_, *f*_source,_ and *f*_general_ to the reference sets of matching, trace‐anchored, source‐anchored, and general match non‐matching scores, respectively. Similar methods were employed in [43]. We use the MATLAB fitdist function to perform kernel density estimation. (Kernel density estimation can be performed in Python with Scikit‐Learn’s Nearest Neighbors library.) The kernel density estimator ${\hat{f}}_{h}:\mathbb{R}\to \left[0,\infty \right)$ applied by fitdist is defined as

(8) $${\hat{f}}_{h}\left(y\right)=\frac{1}{\mathrm{nh}}\sum_{i=1}^{n}\frac{K\left(y-y_{i}\right)}{h},$$

where *n* is the sample size, *y*_1_, …, *y*_n_ are random samples from the unknown distribution, *K* is the kernel smoothing function, and *h* is the bandwidth. In our case, *y*_1_, …, *y*_n_ are the scores, *K* is the normal kernel function [44]. We allow fitdist to choose the optimal bandwidth *h*. Generally, this method of naively selecting the bandwidth will under‐smooth the estimated PDFs because this method relies on the assumption of independent data, which we know we do not have due to the pairwise nature of creating the matching and non‐matching scores. However, there is currently no method of fitting better PDF estimates for pairwise dependent data.

All the pieces are in place for us to calculate the trace‐anchored, source‐anchored, and general match SLRs. The numerator of all three SLR types shown in Equations (4), (5), (6) are estimated by fitting a PDF *f*_m_ to the matching scores matching scores reference set using Equation (8) and evaluating *f*_m_ at the mean similarity score *δ* from Equation (7). The denominator of the trace‐anchored SLR is the PDF *f*_trace_ fit to the set of trace‐anchored non‐matching scores using Equation (8) evaluated at *δ* from Equation (7). The trace‐anchored SLR is defined

$${\mathrm{SLR}}_{\text{trace}}=\frac{f_{m}\left(\delta \right)}{f_{\text{trace}}\left(\delta \right)}.$$

Similarly, the source‐anchored SLR is defined

$${\mathrm{SLR}}_{\text{source}}=\frac{f_{m}\left(\delta \right)}{f_{\text{source}}\left(\delta \right)},$$

where *f*_source_ is the PDF fit to the source‐anchored non‐matching scores. Lastly the general match SLR is defined

$${\mathrm{SLR}}_{\text{general}}=\frac{f_{m}\left(\delta \right)}{f_{\text{general}}\left(\delta \right)},$$

where *f*_general_ is the PDF fit to the general match non‐matching scores.

The SLR_trace_, SLR_source_, and SLR_general_ use the same numerator, which gives the likelihood of observing the score *δ* if *H*_p_ is true. The denominators of the three SLRs give the likelihood of observing the score *δ* if *H*_d_ is true, each SLR type using a different definition of non‐matching scores. An SLR value is the ratio of these two likelihoods. sss 2 shows how SLR values are commonly interpreted based on whether they are less than or greater than 1.

## 3 RESULTS AND DISCUSSION

We calculate the trace‐anchored, source‐anchored, and general match SLRs between 960 questioned images (20 images from each of the 48 camera devices in the dataset) and each of the 48 devices set as the specific known device in turn, resulting in 3 × 960 × 48 = 138,240 SLRs. The prosecution hypothesis *H*_p_ is true (the questioned image *I*_u_ and the camera fingerprint *F*_k_ both originated from the person of interest’s camera *C*_k_) for 3 × 48 × 20 = 2880 of these SLRs, and the defense hypothesis *H*_d_ is true (the fingerprint *F*_k_ originated from the POI’s camera *C*_k_ but the questioned image *I*_u_ did not) for the other 135,360 SLRs.

### 3.1 Overall performance

**FIGURE 7:**
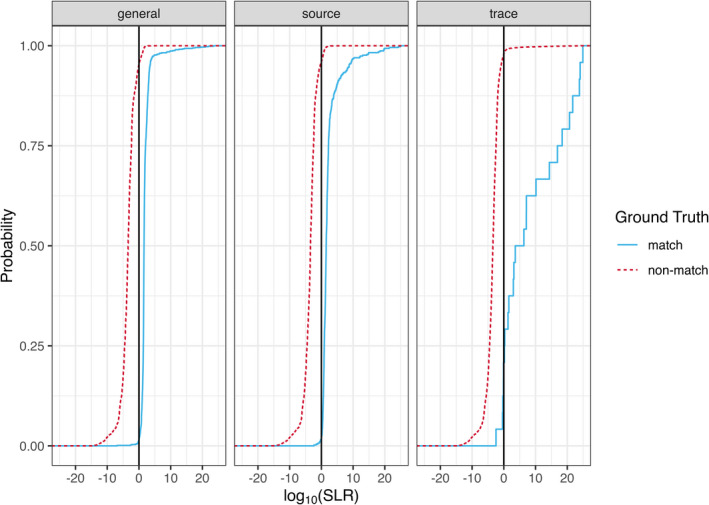
Tippet plots of general match, source‐anchored, and trace‐anchored SLRs under two scenarios: Match (*H*_p_ is true) and non‐match (*H*_d_ is true)

**FIGURE 8:**
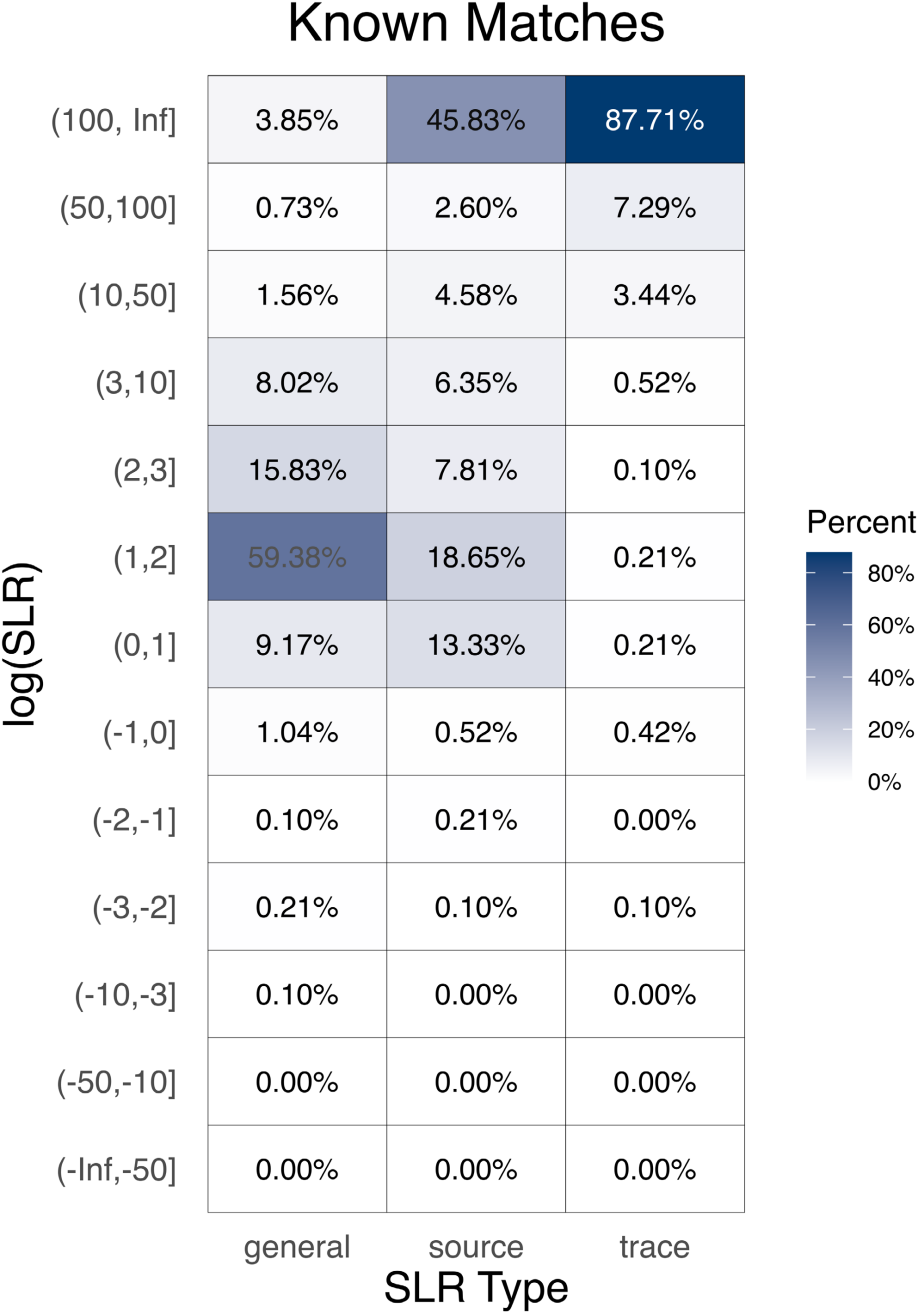
Each tile shows the percentage of known matching log_10_(SLR) values that fall into a particular interval. values greater than 0 correctly support *H*_p_ relative to *H*_d_ and values less than or equal to 0 are misleading evidence in favor of *H*_d_. Values closer to 0 show weaker support and values farther for 0 show stronger support

The Tippet plots in Figure 7 (following the convention used in [45]) show the empirical cumulative distribution function of SLR scores for each SLR type when *H*_p_ is true and when *H*_d_ is true. We see that the three SLR types under consideration perform well but imperfectly. Misleading evidence in favor of *H*_d_ occurs when *H*_p_ is true but the SLR score is less than zero. Misleading evidence in favor of *H*_p_ occurs when *H*_d_ is true but the SLR score is greater than zero. The Tippet plots show that both types of misleading evidence occur for all three SLR types. The trace‐anchored SLRs might appear on first glance to perform poorly when *H*_p_ is true, but that is not the case. The trace‐anchored SLR curve under *H*_p_ rises more slowly than the other two SLR types because the majority of trace‐anchored SLR scores are larger than the source‐anchored and general match scores (see Figure 8). This behavior is precisely what we wish to see because it means that when *H*_p_ is true many of the trace‐anchored SLRs correctly show strong support for *H*_p_ relative to *H*_d_.

The exact rates of misleading evidence in favor of the prosecution (RMEP) and in favor of the defense (RMED) are show in Tables 1 and 2, respectively. Both the RMEP and RMED are lowest for the trace‐anchored SLRs. Tables 3 and 4 show the RMEP and RMED for each camera model. It is interesting to note that a particular model might have its highest RMEP under one SLR type while a different model might have its highest RMEP under a different SLR type. The RMEP is high for some camera models and work should be done to attempt to lower the RMEP. In particular, we propose researching how incorporating an *inconclusive zone* where log_10_(SLR) values close to 0 are considered inconclusive and using *close non‐matches*, cameras of the same model, as the alternative device population might decrease the rates of misleading evidence (see Section 4 for more discussion of future work).

**TABLE 1:** Rates of misleading evidence in favor of *H*_p_

General match	Source‐anchored	Trace‐anchored
0.0466	0.0409	0.0267

**TABLE 2:** Rates of misleading evidence in favor of *H*_d_

General match	Source‐anchored	Trace‐anchored
0.0146	0.00833	0.00521

**TABLE 3:** Rates of misleading evidence in favor of *H*_p_ by the model of the questioned image

Model of questioned image	General match	Source‐anchored	Trace‐anchored
Canon EOS 100D Rebel SL1	0.0277	0.0399	0.0447
Canon EOS 20D	0	0	0
Canon EOS 400D	0	0	0.0011
Canon EOS 60D	0.034	0.0394	0.0404
Canon Rebel XSi	0	0	0.0043
iPad pro 7.1 13 inch	0.183	0.1064	0.0447
iPhone 6s	0.0899	0.0404	0.0149
iPhone 6s Plus	0.0383	0.0117	0.0064
iPhone 7	0.0316	0.0133	0.0106
iPhone 7 Plus	0.05	0.0229	0.0096
iPhone 8	0.0787	0.0489	0.0229
iPhone X	0.0718	0.0495	0.0191
Nikon 1 AW	0	0	0.0011
Nikon D200	0.0191	0.0213	0.0234
Nikon D5200	0.0848	0.0851	0.067
Nikon D70	0.077	0.0848	0.0365
Nikon D70S	0.0819	0.0846	0.0473
Nikon D7100	0.084	0.083	0.0287
OnePlus 5	0.0154	0.0186	0.0218
Panasonic Lumix DMC FZ28	0	0	0.0011
Panasonic Lumix DMC GM1	0	0	0.0011
Pentex K50	0.0213	0.0213	0.0223
Pixel 1	0.0362	0.0574	0.0314
Pixel 2	0.0144	0.0197	0.0202
Samsung Galaxy S8	0.0468	0.0521	0.0617
Sony ILCE alpha 6000	0.0681	0.0809	0.0532

**TABLE 4:** Rates of misleading evidence in favor of *H*_d_ by the model of the questioned image

Model of questioned image	General match	Source‐anchored	Trace‐anchored
Canon EOS 100D Rebel SL1	0.025	0	0
Canon EOS 20D	0	0	0
Canon EOS 400D	0	0	0
Canon EOS 60D	0.05	0.05	0.05
Canon Rebel XSi	0	0	0
iPad pro 7.1 13 inch	0	0	0
iPhone 6s	0	0	0
iPhone 6s Plus	0	0	0
iPhone 7	0.0375	0.0625	0
iPhone 7 Plus	0	0	0
iPhone 8	0.025	0.05	0
iPhone X	0.025	0	0
Nikon 1 AW	0	0	0
Nikon D200	0	0	0
Nikon D5200	0	0	0
Nikon D70	0.05	0	0
Nikon D70S	0	0	0
Nikon D7100	0	0	0
OnePlus 5	0	0	0
Panasonic Lumix DMC FZ28	0	0	0
Panasonic Lumix DMC GM1	0	0	0
Pentex K50	0	0	0
Pixel 1	0.0125	0	0
Pixel 2	0.075	0	0
Samsung Galaxy S8	0	0	0
Sony ILCE alpha 6000	0	0	0.2

### 3.2 Evaluating the strength of the evidence

**FIGURE 9:**
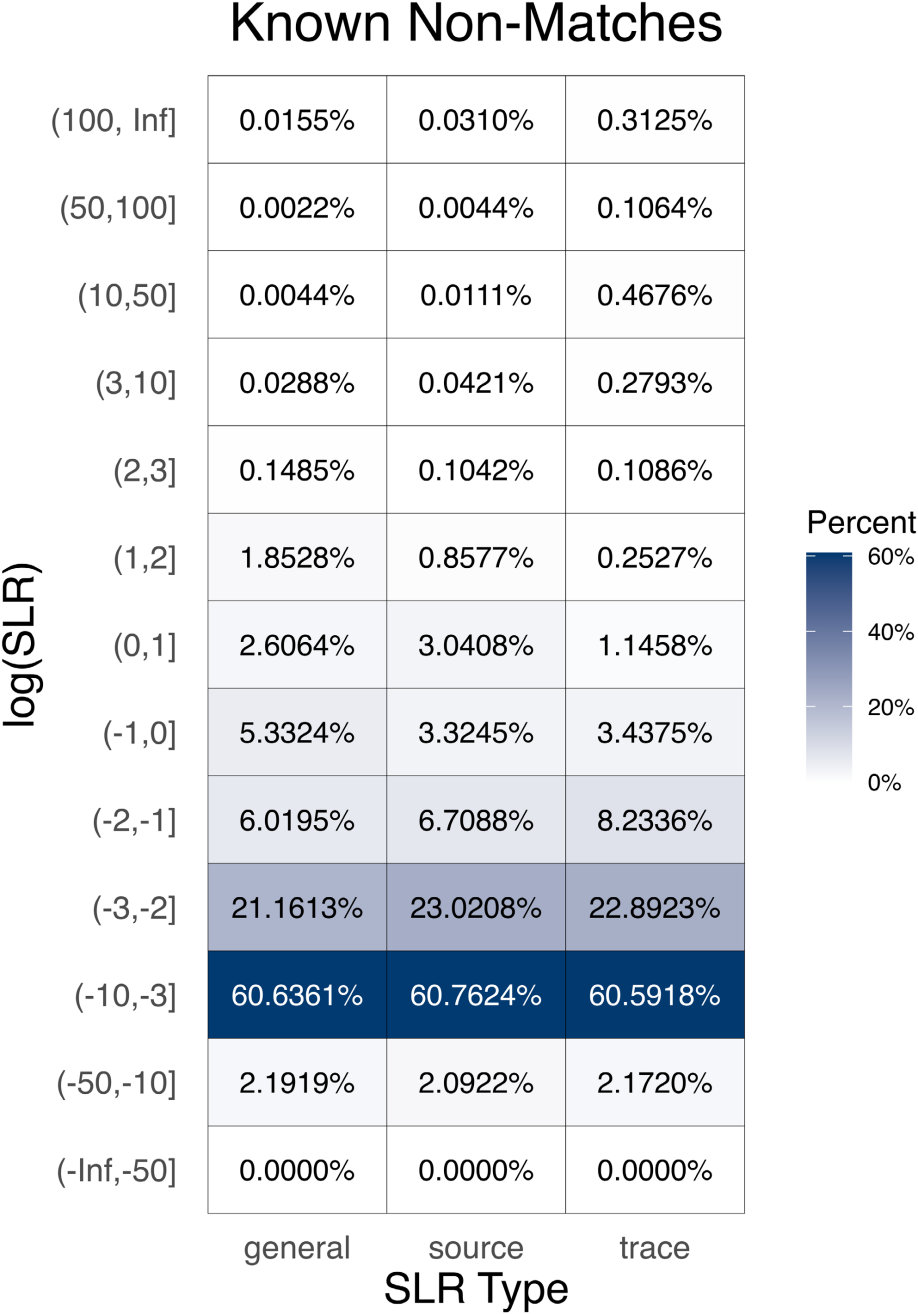
Each tile shows the percentage of known non‐matching log_10_(SLR) values that fall into a particular interval. values less than or equal to 0 correctly support *H*_d_ relative to *H*_p_ and values greater than 0 are misleading evidence in favor of *H*_p_. Values closer to 0 show weaker support and values farther for 0 show stronger support

SLRs allow us to quantify the strength of the evidential support for *H*_p_ or *H*_d_. Figures 8 and 9 group all 138,240 general match, source‐anchored, and trace‐anchored log_10_(SLR) scores into intervals based on their values. Figure 8 shows log_10_(SLR) values from known matches and Figure 9 shows known non‐matches. Each cell displays the percentage of general match, source‐anchored, or trace‐anchored log_10_(SLR) values that fall into a particular interval. Almost 69% of general match SLRs for known matches correctly support *H*_p_ relative to *H*_d_ but the strength of the evidence is rather weak with these values between 0 and 2. On the other hand, 87.71% of the trace‐anchored SLRs for known matches correctly show stronger support for *H*_p_ with log_10_(SLR) values greater than 100, while only 41.83% of source‐anchored SLRs and 3.85% of general match SLRs show the same strength of support for *H*_p_ relative to *H*_d_. In this respect, the trace‐anchored SLRs perform better than the other two SLR types when *H*_p_ is true. It is worth noting that 61.5% of the trace‐anchored SLRs for known matches are infinite because the denominator is zero. This is an artifact caused by KDE. All three SLR types perform well and similarly on known non‐matches. For roughly 10% of the log_10_(SLR) values the evidence in favor of *H*_d_ is rather weak with the values falling between −2 and 0. Over 80% of the log_10_(SLR) values show stronger support for *H*_d_ relative to *H*_p_ with values less than or equal to −2.

## 4 CONCLUSIONS

This paper presents a framework for calculating general match, source‐anchored, and trace‐anchored SLRs to not only address the prosecution’s and defense’s hypotheses in camera device identification problem, but also to provide a means of quantifying the strength of the evidence in favor of one hypothesis over the other. The dataset consists of digital images from 48 camera devices representing 26 distinct models. It includes digital still cameras, mobile phones, and one tablet. To the best of our knowledge, this is the first time camera device identification experiments using all three types of SLRs have been performed. General match SLRs performed poorly on the dataset, while both the source‐anchored and trace‐anchored SLRs performed well, with the trace‐anchored SLRs performing the best of the three. If this dataset were to be used as the reference dataset for camera device identification SLRs in practice, trace‐anchored SLRs would be recommended for use. One large caveat, however, is that it is unknown if similar results would occur on different datasets. Before applying SLRs in practice with a new dataset, it would be a good idea to replicate the study presented in this paper on the new dataset.

We only used RAW, center‐cropped, auto‐exposure, landscape‐oriented images in our dataset. Future research should explore SLRs on a much wider variety of image data. Additionally, we only considered the *closed set* scenario where the questioned image’s camera was present in the dataset. Future work should explore the *open set* scenario where the questioned image’s camera is not in the dataset.

Previous work has shown that it is possible to distinguish between different models based on image artifacts created by the color filter array and the image processing pipeline [46]. We plan to leverage this in future work with SLRs where we restrict the reference dataset to *close non‐matches* where the cameras in the dataset are the same model or brand as the POI’s camera.

Several researchers [25, 47] have employed an *inconclusive zone* where log_10_(SLR) values are considered inconclusive if *t*_1_ < log_10_(SLR) < *t*_2_ for a real‐valued constants *t*_1_ and *t*_2_ where *t*_1_ < *t*_2_. These researchers considered *t*_1_ = −*t* and *t*_2_ = *t* for a constant *t* > 0. This type of inconclusive zone gives equal weight to both hypotheses. Future work could explore methods for choosing the best *t*_1_ and *t*_2_ to minimize the rates of misleading evidence. Additionally, a *defense biased inconclusive zone* with *t*_1_ = 0 and *t*_2_ = *t* for a constant *t* > 0, which places a higher burden of proof on the prosecution while granting the benefit of the doubt to the defense could also be explored.

## ACKNOWLEDGEMENTS

Open access funding provided by the Iowa State University Library.
